# Bacteria and protozoa with pathogenic potential in *Ixodes ricinus* ticks in Viennese recreational areas

**DOI:** 10.1007/s00508-022-02046-7

**Published:** 2022-06-10

**Authors:** Anna-Margarita Schötta, Theresa Stelzer, Gerold Stanek, Hannes Stockinger, Michiel Wijnveld

**Affiliations:** Institute for Hygiene and Applied Immunology, Center for Pathophysiology, Infectiology and Immunology, Medical University of Vienna, Kinderspitalgasse 15, 1090 Vienna, Austria

**Keywords:** Vienna, Borrelia miyamotoi, Tick-borne pathogens, Borrelia, City parks

## Abstract

*Ixodes ricinus* is the most relevant vector for tick-borne diseases in Austria and responsible for the transmission of *Borrelia burgdorferi* sensu lato (s. l.), which causes Lyme borreliosis in humans; however, also other bacteria and protozoa can be found in ticks and have the potential of infecting people and animals. In this study we collected ticks in popular recreational areas in the city of Vienna in the years 2019 and 2020 and analyzed them for the presence of such putative pathogenic microorganisms. By using reverse line blot (RLB) hybridization we detected DNA of *B. burgdorferi* s. l., *Rickettsia* spp., *Babesia* spp., *Candidatus* Neoehrlichia mikurensis (CNM) and *Anaplasma phagocytophilum*. Moreover, we also screened them for the relapsing fever spirochete *Borrelia miyamotoi* employing real-time PCR. The most frequently detected pathogens were *B. burgdorferi* s. l. in 28.6% of the ticks in 2019 and 21.3% of the ticks in 2020. The genus *Rickettsia* was detected in 13.8% of the ticks from 2019 and only in 4.6% from 2020. *Babesia* spp. were detected in 5.7% in 2019 and 4.2% in 2020. Furthermore, we detected CNM in 4.0% (2019) and 5.6% (2020), *A. phagocytophilum* in 0.5% (2019) and 1.3% (2020) and finally *B. miyamotoi* in 3.3% (2019) and 1.7% (2020). Collectively, we show that various microorganisms are prevalent in ticks collected in Vienna and identify hotspots for *B. miyamotoi,* which we have detected for the first time in the city.

## Introduction

*Ixodes ricinus* is the most abundant tick species in Austria and is commonly associated with the transmission of a large number of (potentially) pathogenic microorganisms [1–3]. Out of the large number of tick-borne pathogens, spirochetes of the *Borrelia burgdorferi* sensu lato (s. l.) complex, the causative agent for Lyme borreliosis, are the most frequently found pathogens in *Ixodes *ticks collected in Austria [4, 5]. While the genospecies *B. afzelii, B. burgdorferi* sensu stricto (s.s.), *B. garinii, B. bavariensis* and *B. spielmanii* are known to cause disease in humans, the genospecies *B. bissettii, B. lusitaniae*, and *B. valaisiana *are believed to be of lower importance for human disease [6, 7]. The different possible manifestations of the disease have been linked to different genospecies: *B. afzelii* is known to cause primarily skin manifestations, *B. garinii* and *B. bavariensis* are more frequently linked to neuroborreliosis and *B. burgdorferi* s.s. seems to affect the joints more than other genospecies [8].

Aside from *B. burgdorferi* s. l., *Anaplasma phagocytophilum, Candidatus *Neoehrlichia mikurensis and several *Babesia* and *Rickettsia *species have been detected in *Ixodes* ticks collected in Austria in the past [5, 9, 10].

The intracellular bacterium *A. phagocytophilum* is known to infest the granulocytes and can lead to flu-like illness, so-called human granulocytic anaplasmosis (HGA), a self-limiting infection in most cases but leading to severe and fatal disease in immunocompromised patients [11–13].

An infection with *Candidatus* N. mikurensis can cause symptoms, such as fever, malaise, weight loss and septicemia by affecting the vascular endothelium [14–17]; however, recently we showed that this pathogen can persist multiple weeks in human blood without causing any symptoms [18].

The intraerythrocytic parasites of the genus *Babesia* belong to the phylum of Apicomplexa and can lead to babesiosis, a flu-like to malaria-like illness including symptoms like malaise, chills, myalgia, anemia, fatigue and fever [19]. In immunocompromised, especially in splenectomised, individuals *Babesia* infections can lead to life-threatening complications like severe hemolysis [19]. *B. venatorum* infection of an immunocompromised 56-year-old male, most likely caused by a tick bite, was reported in Austria in 2003 [20]. Also, human infections with the species *Babesia divergens* and *Theileria* (*Babesia*)* microti* have already been reported in Europe [21–23].

Within the genus *Rickettsia* the spotted-fever group (SFG) species *R. helvetica, R. monacensis, R. raoultii *and* R. slovaca* have been detected in ticks collected in Austria [5, 10]. The species *R. slovaca* and *R. raoultii *are known to cause scalp eschar, facial edema and cervical lymphadenopathy, the so-called tick-borne lymphadenopathy (TIBOLA) or Dermacentor-borne necrosis erythema and lymphadenopathy (DEBONEL) [24]. The infection with *R. helvetica* has been occasionally associated with perimyocarditis and eruptive fever in the literature but other studies showed that despite a high prevalence in ticks, *R. helvetica* poses no risk to humans [18, 25, 26]. *R. monacensis* infections present with symptoms like fever, headache, general discomfort, joint pain and erythematous rash [27]. Non-SFG *Rickettsiae*, specifically *Candidatus* R. mendelii and *Candidatus* R. thierseensis, have been found in ticks in Austria as well [18, 28].

The relapsing fever spirochete* Borrelia miyamotoi* has been detected in *I. ricinus* ticks collected in Austria during the year 2005 [29] but not in Vienna. The disease caused by *B. miyamotoi* infection is described as a nonspecific febrile illness which can also occur asymptomatically and human cases have sporadically been reported in Austria [18, 30, 31].

Due to this large variety of pathogens known to be present in *I. ricinus* in Austria and the fact that urban recreational areas in Vienna are regularly visited by the citizens, the infection rate of ticks in these areas is of particular medical interest. To assess this, we collected questing *I. ricinus* ticks at seven popular green spaces in Vienna and screened them for a large panel of potentially pathogenic microorganisms by using the reverse line blot hybridization assay (RLB) and real-time PCR.

## Material and methods

### Collecting of ticks

**Fig. 1:**
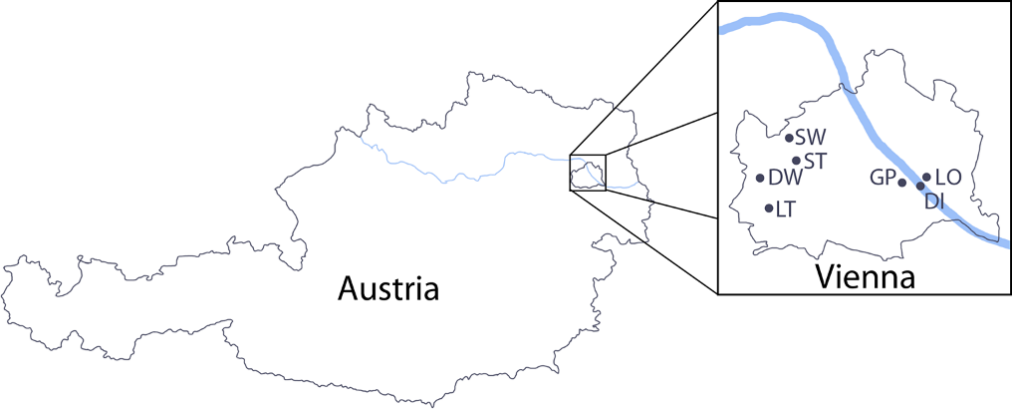
Tick collection sites in Vienna. Schematic map of Austria with magnification of Vienna showing the seven collection sites. *DW* Deutschordenswald, *DI* Donauinsel, *GP* Grüner Prater, *LT* Lainzer Tiergarten, *LO* Nationalpark Lobau, *SW* Schottenwald, *ST* Steinhofgründe. (The figure is modified from Google Maps, https://maps.google.com/)

From April to May 2019 questing ticks were collected at Deutschordenswald (48°12′02.6″N 16°13′38.1″E), Donauinsel (48°11′14.3″N 16°28′14.2″E), Grüner Prater (48°11′30.7″N 16°26′11.8″E), Lainzer Tiergarten (48°10′18.5″N 16°14′58.4″E), Nationalpark Lobau (48°11′37.0″N 16°28′19.9″E), Schottenwald (48°14′05.3″N 16°16′04.4″E) and Steinhofgründe (48°12′38.2″N 16°16′32.9″E) (Fig. 1). From May to July 2020 questing ticks were collected again in the same areas with the exception of Schottenwald (due to SARS-CoV‑2 limitations in time and resources). All ticks were collected by dragging a white cotton cloth through the sampling area. The collection sites showed similar features like mixed forests with strong undergrowth, meadows, and wildlife. For tick collection sunny to cloudy, but dry days were chosen and the temperature during sampling varied from 17 °C to 24 °C in 2019 and from 14 °C to 29 °C in 2020.

### Tick identification and DNA extraction

Prior to DNA extraction, the *I. ricinus* ticks were morphologically identified using a current identification key [32]. No additional molecular identification of the collected ticks was carried out and we considered all screened *I. ricinus* ticks to belong to the *I. ricinus* complex which includes *I. inopinatus,* due to the recently observed inability to distinguish *I. ricinus* from *I. inopinatus* molecularly [33]. In 2019, from all locations 30 ticks were tested. In 2020, 40 ticks were tested from every location except from Schottenwald where no sampling took place in 2020. Our study was focused on nymphal ticks as this is the life stage of the highest medical relevance to humans. In case not enough nymphal ticks were flagged we added adult ticks to reach a minimum number of 30 ticks per location. In 2020 exclusively nymphs were investigated. In 2019 we included some adult ticks for the locations Donauinsel (*n* = 10), Schottenwald (*n* = 2), Steinhof (*n* = 2) and Deutschordenswald (*n* = 10) to reach 30 ticks per collection site.

The DNA was extracted using the DNeasy Blood and Tissue Kit (Qiagen, Hilden, Germany). Briefly, individual ticks were washed in 70% ethanol, air-dried and cut into halves using a sterile surgical blade. The lysis step was performed by overnight incubation at 56 °C in 180 µL ATL buffer and 20 µL proteinase K. The following DNA extraction steps were performed according to the manufacturer’s protocol. DNA was eluted with 100 µL pre-warmed (70 °C) AE buffer and stored at −20 °C until further use.

### Pathogen detection by RLB

Genus-specific PCR targeting the *Anaplasma/Ehrlichia/Neoehrlichia *16S rRNA gene, the *Babesia/Theileria *18S rRNA gene, the *Borrelia *23S-5S intergenic spacer (IGS), the *Rickettsia *16S rRNA gene and the *Rickettsia *23S-5S IGS were performed for each tick DNA extract using a 5’biotin-labeled primer per PCR reaction as described elsewhere [5, 34]. The RLB was performed according to a protocol described previously [35]. Briefly, the PCR products were combined for each tick individually in 2x SSPE buffer (Thermo Scientific, Vienna, Austria) consisting of 0.3M NaCl, 0.02 NaH_2_PO_4,_ 0.002 M EDTA and additionally added 0.1% sodium dodecyl sulphate (SDS, Applichem, Darmstadt, Germany). The combined PCR products were denatured and the single-stranded PCR products were then hybridized to a nylon membrane (Biodyne C, Pall Laboratories, Crailsheim, Germany) with a set of covalently linked genus-specific (catch-all) and species-specific oligonucleotide probes (Eurofins Genomics GmbH, Ebersberg, Germany) allowing the detection of several *Borrelia *spp., *Rickettsia *spp., *Babesia *spp.,* Anaplasma *and *Ehrlichia* spp. (individual probes used are listed by Schötta et al. 2017 [5]). After incubation with 1:10,000 diluted horseradish peroxidase-labelled streptavidin (Roche Diagnostics GmbH, Mannheim, Germany) visualization of the hybridized PCR products was enabled by the Pierce ECL Western blotting substrate (Thermo Fisher Scientific, Vienna, Austria) and the ChemiDoc touch imaging system (Bio-Rad, Hercules, CA, USA). For quality control of the PCR step, DNA extracts of bacterial cultures (*B. burgdorferi *s.s*., B. afzelii, R. raoultii*) or DNA extracts of confirmed-positive ticks (*A. marginale, B. canis, C.* N. mikurensis) were used. For the quality control of the blotting step, pooled biotinylated PCR amplicons of confirmed-positive ticks (*A. phagocytophilum, T.* (*B.*) *microti, B. afzelii, R. monacensis, R. raoultii, R. slovaca*) were used.

### Detection of *B. miyamotoi* by real-time PCR

To detect *B. miyamotoi* DNA, we used a real-time PCR targeting the glycerophosphodiester phosphodiesterase gene (*glpQ*) as published by Reiter et al. 2015 [29]. The PCR reaction (20 µL total volume) contained 10 µl 2 × GoTaq® Probe qPCR Master Mix (Promega), 0.5 µM of each primer, 0.25 µM probe (Sigma-Aldrich, Vienna, Austria) and 4 µl of DNA. The reaction volume was adjusted with PCR-grade water (Sigma-Aldrich, Vienna, Austria). A two-step real-time PCR was run in an Applied Biosystems™ (Thermo Fisher Scientific, Vienna, Austria) QuantStudio 5 Real-Time PCR system with the following conditions: 95 °C for 10 min followed by 50 cycles of 95 °C for 15 s and 60 °C for 1 min. One reaction using a plasmid containing the target sequence and one reaction using PCR-grade water instead of DNA were included for quality control of the PCR run.

### Statistical analysis

IBM SPSS Statistics 25.0 was used to analyze the collected dataset by descriptive statistics. Differences within nominal categories (e.g., tick life stage, infection rate) and the significance of co-infections were calculated using Fisher’s exact test or Pearson’s χ^2^-test. Two-tiered *p*-values of < 0.05 were considered significant.

## Results

### *Borrelia burgdorferi* sensu lato

In 2019, of the 210 ticks 60 (28.6%) and in 2020 of the 240 ticks 51 (21.3%) were positive for at least 1 member of the *B. burgdorferi* s. l. complex by RLB. The most common genospecies detected in 2019 as well as in 2020 was *B. afzelii* (16.2% in 2019; 15.0% in 2020), followed by *B. burgdorferi* s. s. (6.7% in 2019; 3.8% in 2020; Table 1).

**Table 1:** Microorganisms detected in ticks in 2019 and 2020

Year of collection	Location	No. of ticks	*B. burgdorferi* s. l.					*Rickettsia* spp		*Babesia *spp		Others		
			Ba	Bbss	Bg/Bbav	Bsp	Bval	Rh	Rm	Bv	T(B)m	Ap	CNm	Bomy
2019	Deutschordenswald	20 N, 10 A	2 N, 4 A	2 A	3 A	–	1 A	2 N	–	**–**	1 N, 1 A	–	3 N, 2 A	1 A
	Donauinsel	20 N, 10 A	3 N, 1 A	1 N	3 N	1 N, 2 A	4 A	1 N, 4 A	1 A	**–**	1 N	–	–	1 N
	Grüner Prater	30 N	2	5	1	–	–	7	–	**–**	–	1	–	–
	Lainzer Tiergarten	30 N	10	–	–	–	–	2	–	–	7	–	4	4
	Lobau	30 N	2	3	2	–	–	7	–	**–**	–	–	1	1
	Schottenwald	28 N, 2 A	2 N	–	–	–	–	2 N, 1 A	–	–	1 N	–	2 N	–
	Steinhof	28 N, 2 A	7 N, 1 A	3 N	–	–	–	2 N	–	–	1 N	–	1 N, 1 A	–
2020	Deutschordenswald	40 N	6	–	1	–	1	2	–	–	–	1	3	–
	Donauinsel	40 N	6	3	1	–	1	1	1	1	–	–	–	–
	Grüner Prater	40 N	3	2	–	–	–	1	–	1	1	1	1	–
	Lainzer Tiergarten	40 N	9	–	–	–	2	4	1	1	4	1	4	1
	Lobau	40 N	5	2	1	–	1	1	–	–	1	–	–	1
	Steinhof	40 N	7	2	–	–	–	–	–	–	1	–	–	2

*A* adults, *N* nymphs, *Ba B. afzelii, Bbss B. burgdorferi* s.s., *Bg/Bbav B. garinii*/*B. bavariensis, Bsp B. spielmanii, Bval B. valaisiana, Rh R. helvetica, Rm R. monacensis, Bv B. venatorum, T(B)m T. (B.) microti, Ap A. phagocytophilum, CNm Candidatus Neoehrlichia mikurensis, Bomy B. miyamotoi*

We detected *B. garinii/B. bavariensis *DNA in 9 of the 210 (4.3%) ticks from 2019 and in 3 of the 240 ticks (1.3%) collected in 2020. *B. spielmanii* was detected in 3 of the 210 ticks (1.4%) from 2019 and was not detected in any of the ticks collected in 2020, 5 of the 210 ticks (2.4%) from 2019 and 5 of the 240 ticks (2.0%) from 2020 were positive for *B. valaisiana*. *B. spielmanii* was only found in three ticks collected at Donauinsel in 2019. The highest number of *B. afzelii* positive ticks was found in both years at the location Lainzer Tiergarten. In 2019, no other genospecies of the *Borrelia burgdorferi* s. l. complex was detected at Lainzer Tiergarten; in 2020 we also detected *B. valaisiana *in 2 of the 40 ticks. In ticks collected at Steinhof we detected only the genospecies *B. afzelii *and *B. burgdorferi* s. s. in both years. Ticks from Schottenwald were only collected in 2019 and we detected *B. afzelii* in 2 out of 30 ticks. Co-infections with multiple *Borrelia burgdorferi* s. l. species occurred in four ticks, of which two were detected in nymphs (Table 2).

**Table 2:** All single microorganisms and co-infections detected within the collected ticks

Species	No. (%) of infected ticks 2019 (*n* = 210 *n*_(+)_ = 97)	No. (%) of infected ticks 2020 (*n* = 240 *n*_(+)_ = 71)
Single infections	***n*** **=** **72**	***n*** **=** **58**
*B. afzelii*	18 (8.6)	25 (10.4)
*B. burgdorferi s.s.*	8 (3.8)	7 (2.9)
*B. garinii/B. bavariensis*	7 (3.3)	2 (0.8)
*B. spielmanii*	2 (1.0)	5 (2.1)
*B. valaisiana*	4 (1.9)	6 (2.5)
*R. helvetica*	20 (9.5)	2 (0.8)
*R. monacensis*	1 (0.5)	2 (0.8)
*T.* (*B.*) *microti*	1 (0.5)	2 (0.8)
*A. phagocytophilum*	1 (0.5)	3 (1.3)
*Cand*. N. mikurensis	7 (3.3)	3 (1.3)
*B. miyamotoi*	3 (1.4)	1 (0.4)
Dual infections	***n*** **=** **20**	***n*** **=** **10**
*B. afzelii* + *T.* (*B.*)* microti*	6 (2.9)	2 (0.8)
*B. afzelii* + *B. burgdorferi*	2 (1.0)	2 (0.8)
*B. afzelii* *+* *Cand*. N. mikurensis	–	2 (0.8)
*B. afzelii* + *R. helvetica*	2 (1.0)	1 (0.4)
*T.* (*B.*)* microti* *+* *Cand*. N. mikurensis	2 (1.0)	–
*B. burgdorferi* s.s. + *R. helvetica*	1 (0.5)	–
*B. burgdorferi* s.s. + *B. garinii/bavariensis*	1 (0.5)	–
*B. spielmanii* + *R. helvetica*	1 (0.5)	–
*B. valaisiana* *+* *R. helvetica*	1 (0.5)	–
*B. afzelii* *+* *Cand*. N. mikurensis	1 (0.5)	–
*R. helvetica* *+* *Cand*. N. mikurensis	1 (0.5)	–
*B. afzelii* *+* *B. miyamotoi*	–	1 (0.4)
*B. burgdorferi *s.s. + *B. miyamotoi*	1 (0.5)	–
*B. garinii/bavariensis* *+* *B. miyamotoi*	–	1 (0.4)
*T.* (*B.*)* microti* *+* *B. miyamotoi*	1 (0.5)	–
*Cand*. N. mikurensis *+* *B. venatorum*	–	1 (0.4)
Triple infections	***n*** **=** **4**	***n*** **=** **2**
*B. afzelii* *+* *Cand*. N. mikurensis *+* *R. helvetica*	1 (0.5)	–
*B. afzelii* *+* *T.* (*B.*)* microti* *+* *R. helvetica*	1 (0.5)	1 (0.4)
*B. afzelii* *+* *B. miyamotoi* *+* *Cand*. N. mikurensis	1 (0.5)	–
*B. afzelii* *+* *B. burgdorferi* s.s. *+* *B. garinii/bavariensis*	1 (0.5)	–
*B. afzelii* *+* *T.* (*B.*)* microti* *+* *Cand*. N. mikurensis	–	1 (0.4)
Quadruple infections	***n*** **=** **1**	***n*** **=** **0**
*B. afzelii* + *T.* (*B.*)* microti* + *B. miyamotoi* + *Cand*. N. mikurensis	1 (0.5)	–
Quintuple infections	***n*** **=** **0**	***n*** **=** **1**
*B. afzelii* *+* *T.* (*B.*)* microti* *+* *B. miyamotoi* *+* *Cand*. N. mikurensis *+* *R. helvetica*	–	1 (0.4)

Of the 210 ticks from 2019, 24 (11.4%) ticks were adult ticks of which 15 (62.5%) were positive for *Borrelia burgdorferi* s. l., hence, contributing to a significant number of positive ticks. A detailed overview with respect to the tick life stage is shown in Table 1.

### Rickettsia species

Of the 210 ticks 29 (13.8%) collected in 2019 and 11 of the 240 ticks (4.6%) from 2020 were positive for *Rickettsia* species (Table 1); in both years only the species *R. helvetica *(13.3% in 2019; 3.8% in 2020) and *R. monacensis* (0.5% in 2019; 0.8% in 2020) were detected. No tick individual was co-infected with two *Rickettsia* species. In 2019, we detected *R. helvetica* DNA in ticks from every location and in 2020 we detected *R. helvetica* at Deutschordenswald, Donauinsel, Grüner Prater, Lainzer Tiergarten and Lobau, but in none of the 40 ticks collected at Steinhof.

Among nymphs the *Rickettsia* infection rate was 12.4% (23/186) in 2019 and 4.6% (11/240) in 2020; among adults the infection rate in 2019 was 25% (6/24).

### *Babesia* species

Of the 210 ticks 12 (5.7%) collected in 2019 and 10 of 240 ticks (4.2%) collected in 2020 were positive for *Babesia *spp. (Table 1). All positive ticks from the 2019 collection and 7 positive ticks from the 2020 collection were infected with the species *T.* (*B.*) *microti*; 3 positive ticks from 2020 were infected with *B. venatorum*. Out of the 12 *Babesia* positive ticks 7 (58.3%) from 2019 and 5 out of the 10 *Babesia* positive ticks (50%) from 2020 were collected at the location Lainzer Tiergarten. We did not detect *B. venatorum *in the 210 ticks collected in 2019. In 2020 we detected *B. venatorum* in 1 of each of the 40 nymphs collected at Donauinsel, Grüner Prater and Lainzer Tiergarten. Among ticks in the nymphal life stage the *Babesia *spp*.* infection rate was 5.9% (11/186) in 2019 and 4.1% (10/240) in 2020; among adult ticks collected in 2019 the infection rate was 4.3% (1/23).

### *Anaplasma* and *Ehrlichia* species

In 1 of the 210 ticks (0.5%) collected in 2019 and in 3 of the 240 ticks (1.3%) collected in 2020 we detected *A. phagocytophilum* (Table 1). In both years one positive tick was collected at the location Grüner Prater and in 2020 one additional positive tick was collected each at the location Lainzer Tiergarten and at Deutschordenswald. Among the nymphs the infection rate was 0.5% in 2019 and no adult tick from 2019 tested positive for *A. phagocytophilum*. For *Cand*. *N. mikurensis* 8 of the 210 ticks (4.0%) from 2019 and 14 of the 240 ticks (5.8%) from 2020 were positive*.* Among the nymphs from 2019 the infection rate was 6.0% and among adult ticks the infection rate was 12.5% in 2019.

### *Borrelia miyamotoi*

We detected *B. miyamotoi *DNA by real-time PCR in 7 out of the 210 ticks (3.3%) collected at the locations Deutschordenswald, Donauinsel, Lainzer Tiergarten and Lobau in 2019 and in 4 of the 240 ticks (1.7%) collected at the locations Lainzer Tiergarten, Lobau and Steinhof in 2020 (Table 1). We collected the highest number of *B. miyamotoi* positive ticks in 2019 at Lainzer Tiergarten where 4 out of 30 ticks (13.3%) were positive.

Among nymphs, an infection rate of about 3.2% was calculated in 2019 and 1.6% in 2020. In adult ticks collected in 2019, the infection rate was 4.2%.

### Detection of infections and co-infections in *Ixodes ricinus* ticks

Of the 210 ticks 97 (46.2%) collected in 2019 and 71 of the 240 ticks (29.6%) collected in 2020 tested positive for at least 1 pathogenic species (Table 2). In 2019, 72 ticks (34.8%) were infected with 1 pathogen, 20 ticks (9.5%) showed a dual infection, 4 ticks (1.9%) harboured 3 and one 1 (0.5%) harbored 4 pathogenic species; from the 2020 collection 58 of the 240 ticks (24.2%) were positive for 1 species, 10 (4.2%) for 2 species, 2 (0.8%) for 3 species and in 1 nymph (0.4%) we detected 5 species (Table 2). Co-occurrences of tick-borne pathogens were investigated with the Fisher’s exact test. In both years, detection of the co-occurrence was significant for *Babesia *spp. and *Cand*. N. mikurensis (2019: *P* = 0.036; 2020: *P* = 0.003) and *B. burgdorferi *s. l. and *Babesia* spp. (2019: *P* = 0.005, 2020: *P* = 0.038). In 2020, the co-occurrence of *B. burgdorferi* s. l. and *B. miyamotoi* was also significant (*P* = 0.031).

## Discussion

In this study, we determined the tick infection rates for numerous tick-borne pathogens in urban city parks and suburban forests of Vienna. A total of 450 ticks (426 nymphs, 24 adults) were collected during the summer months at 7 (sub)urban locations and screened by RLB, a DNA-hybridization method which is feasible for a reliable detection of various tick-borne pathogens on genus and species levels at once. Moreover, we also investigated the ticks of Vienna for the presence of the relapsing fever spirochete *B. miyamotoi* by real-time PCR. The focus of this study was laid on nymphs, as this life stage of *I. ricinus* is the medically most relevant stage when it comes to transmitting a pathogen that could cause disease in humans. Next to their small size and thus easily overlooked while feeding, nymphs already underwent one previous bloodmeal during the larval stage and therefore might have taken up microorganisms from the host blood which could now be transmitted during their second blood meal. A recent study conducted in Austria showed that 72.1% of the reported human tick bites are ticks of the nymphal life stage [18].

With 28.6% positive ticks (60/210) in 2019 and 21.3% (51/240) in 2020, *B. burgdorferi *s. l. was the most common pathogen detected. The percentages calculated lie within the range shown in studies carried out previously in Austria and surrounding countries [5, 36, 37]. Within the *B. burgdorferi* s. l. complex, the most abundant genospecies was *B. afzelii*. As mentioned in the introduction, *B. afzelii* is the main cause of skin manifestations such as erythema migrans; however, in rare cases, *B. afzelii* can mimic a rickettsial infection by causing a scalp eschar [38]. Furthermore, in a recent case report, it was noted that *B. afzelii* DNA was detected in ocular tissue [39] making *B. afzelii* one of the most medically significant borrelial species.

The second most common genus detected over our 2‑year survey was *Rickettsia.* In 2019, 14.3% out of all ticks tested positive for *Rickettsia* spp. Strikingly, in 2020 only 4.6% of the ticks tested positive for *Rickettsia* spp. Next to the *Rickettsia* species *R. helvetica* and *R. monacensis*, which were detected in both years at several of our locations, no other species were detected even though species like *R. raoultii, R. slovaca* and the novel *Candidatus* R. thierseensis are known to be present in Austrian *I. ricinus* ticks [5, 28]. The finding that only *R. helvetica* and *R. monacensis* were found in this study might be due to their higher prevalence and distribution compared to the other genospecies.

The tick infection rates for *B. miyamotoi, Babesia *spp., *A. phagocytophilum* and *Candidatus *N. mikurensis were beneath 6.0% in both years and lie within the range previously described [5, 29, 40–42]. An astonishing finding was the significant co-occurrence of *Babesia* spp. and *Candidatus* N. mikurensis which was also already seen in a previous investigation of Austrian ticks [5]. It might be of great interest to further study the interaction of different microorganisms within the tick vector and whether this might have an influence on uptake of pathogens or transmission dynamics.

For both years, the location Lainzer Tiergarten was identified as a ‘hotspot’ for *B. afzelii*. In 2019 at Lainzer Tiergarten, 33.3% of the collected ticks were positive for *B. afzelii* and no other *B. burgdorferi* s. l. genospecies were detected. In 2020, 22.5% of the ticks were positive for *B. afzelii *(81.8% of the *Borrelia burgdorferi* sl positive ticks). Aside from *B. afzelii*, Lainzer Tiergarten was also identified as a ‘hotspot’ for the relapsing fever spirochete* B. miyamotoi* in 2019 with a tick infection rate of 13.3%, compared to the overall infection rate of 3.3% in 2019. In 2020, only 2.5% of the ticks collected at Lainzer Tiergarten were infected with this relapsing fever spirochete, but this is also above the 1.7% calculated among all locations in 2019. Furthermore, 58.3% of the *Babesia* spp. detected in 2019 and 50.0% of the *Babesia *spp. detected in 2020 were collected at Lainzer Tiergarten. Identifying hotspots is not just of epidemiological interest but also aids further studies of certain tick-borne pathogens when it comes to cultivation attempts and where to best collect ticks for certain experiments.

The Lainzer Tiergarten, a 2450 hectar Natura 2000 area (the largest coordinated network of protected areas in the world), was originally a fenced off hunting ground used by the Austrian imperial family. In 1919, the region was opened to the public and since 1941 has been classified as a nature reserve. The area supports a large diversity of wildlife, and as currently announced on the website (https://www.lainzer-tiergarten.at/ visited on the 19 January 2022), the Lainzer Tiergarten estimates to host, among other animals, 800–1000 wild boars, 80–100 red deer, 200–250 fallow deer, countless roe deer and around 700 mouflons. We suspect that the large density and diversity of wildlife present plays a large role in creating an unintended hotspot for tick-borne pathogens.

In conclusion, our study emphasizes the need to keep track of ticks and tick-borne pathogens within urban recreational areas to early identify potential health risks.
